# Pharmacological Profiling of Purified Human Stem Cell-Derived and Primary Mouse Motor Neurons

**DOI:** 10.1038/s41598-019-47203-7

**Published:** 2019-07-25

**Authors:** Daniel Moakley, Joan Koh, Joao D. Pereira, Daniel M. DuBreuil, Anna-Claire Devlin, Eugene Berezovski, Kevin Zhu, Brian J. Wainger

**Affiliations:** 1 Department of Neurology, Massachusetts General Hospital, Harvard Medical School, Boston, MA 02114 USA; 2 Department of Anesthesiology, Critical Care and Pain Medicine, Massachusetts General Hospital, Boston, MA 02114 USA; 3 Harvard Stem Cell Institute, Cambridge, MA 02138 USA; 4 Broad Institute of Harvard University and MIT, Cambridge, MA 02142 USA

**Keywords:** Excitability, Stem-cell biotechnology

## Abstract

Directed differentiation of human pluripotent stem cells (hPSCs) has enabled the generation of specific neuronal subtypes that approximate the intended primary mammalian cells on both the RNA and protein levels. These cells offer unique opportunities, including insights into mechanistic understanding of the early driving events in neurodegenerative disease, replacement of degenerating cell populations, and compound identification and evaluation in the context of precision medicine. However, whether the derived neurons indeed recapitulate the physiological features of the desired *bona fide* neuronal subgroups remains an unanswered question and one important for validating stem cell models as accurate functional representations of the primary cell types. Here, we purified both hPSC-derived and primary mouse spinal motor neurons in parallel and used extracellular multi-electrode array (MEA) recording to compare the pharmacological sensitivity of neuronal excitability and network function. We observed similar effects for most receptor and channel agonists and antagonists, supporting the consistency between human PSC-derived and mouse primary spinal motor neuron models from a physiological perspective.

## Introduction

Deriving specific disease-relevant cellular subtypes from human pluripotent stem cells (hPSCs) may serve potentially valuable roles in disease modelling, cell replacement, and drug development^1^. The approach holds promise both for individual diseases and for subgroups as may be pursued in personalized or targeted medicine^2^. The potential value for such applications is particularly large in neurodegenerative diseases, for which access to early stage tissue is difficult or impossible and for which the large majority of disease cases are apparently sporadic, potentially limiting the accuracy of mouse models when mechanistic inference from rare monogenetic cases may not extend to sporadic ones. Furthermore, a growing appreciation for the effects of human-specific genes and genetic background adds additional levels of complexity that cannot be addressed in mouse models^3,4^.

Initial hPSC modeling studies identified neuronal subtypes such as spinal motor neurons based on the expression of specific protein markers^5^, and more recent reports have used unbiased RNA expression profiles to support the accuracy of the model motor neurons^6^. While the RNA transcriptome yields a valuable and unbiased view of the templates available for cellular function, differences in RNA processing, spatial restrictions, translation, and post-translational modification may yield profound differences in the integrated ensemble of cellular functions^7^. Indeed, the primary role of a neuron is a physiological one: to integrate synaptic inputs and generate an efferent signal transmitted to another neuron or effector cell such as muscle in the case of spinal motor neurons. How accurately stem cell models reflect the physiology of *bona fide* neurons remains unaddressed.

The rich diversity of voltage-gated ion channels, particularly potassium channels, and the specific combinations of these channels enable fine tuning of the physiological features of neuronal subpopulations to match and in some cases even define their functions^8^. Thus, focusing on physiology and particularly the cohort of functional ion channels in specific neurons can provide a parallel functional assessment of the value of human stem cell-based models.

Recent clinical phenotypes have raised the question as to whether abnormal neuronal excitability contributes to specific neurological diseases and whether the physiological features and functions of neuronal populations may contribute to their selective vulnerabilities. This possibility seems particularly strong in familial epilepsies and rare pain syndromes due to mutations in specific sodium and potassium channels^9^. However, a growing body of evidence supports this position in multiple neurodegenerative diseases as well, including amyotrophic lateral sclerosis (ALS)^10–12^, Parkinson’s disease^13^, and Alzheimer’s disease^14,15^. While a group of stem cell modelling studies have already documented abnormal motor neuron excitability in ALS, the underlying assumption that the physiology of hPSC-derived motor neurons accurately reflects the physiology of *bona fide* motor neurons has not been addressed. Thus, the physiological concordance of the model neurons with primary ones may be particularly important in modelling diseases of specific vulnerable neuronal types.

To assess the similarity of hPSC-derived spinal motor neurons and *bona fide* primary motor neurons and identify major determinants of excitability in each group, we compared the pharmacological profiles of purified human and mouse motor neurons using multi-electrode array (MEA) recordings. We observed a high degree of similarity between motor neurons derived from hPSCs and mice using immunocytochemistry and qPCR of ion channel transcripts. We then quantified the spontaneous activity of motor neuron cultures from each species using longitudinal MEA recording over a span of four weeks. Using a timepoint at which both mouse and human motor neuron cultures exhibited robust network activity, we first determined the contribution of multiple neurotransmitters to synchronized neuronal firing. We then identified the contributions of individual ion channel classes to intrinsic excitability and action potential firing. Although the effect sizes were sometimes larger in primary mouse motor neuron cultures than in hPSC-derived motor neuron cultures, consistent with a greater functional maturation of the primary neurons, the neurotransmitter and ion channel blockers controlling motor neuron networking and intrinsic firing were similar in both groups.

## Results

### Preparation of hPSC-derived and primary mouse spinal motor neuron cultures

**Figure 1:**
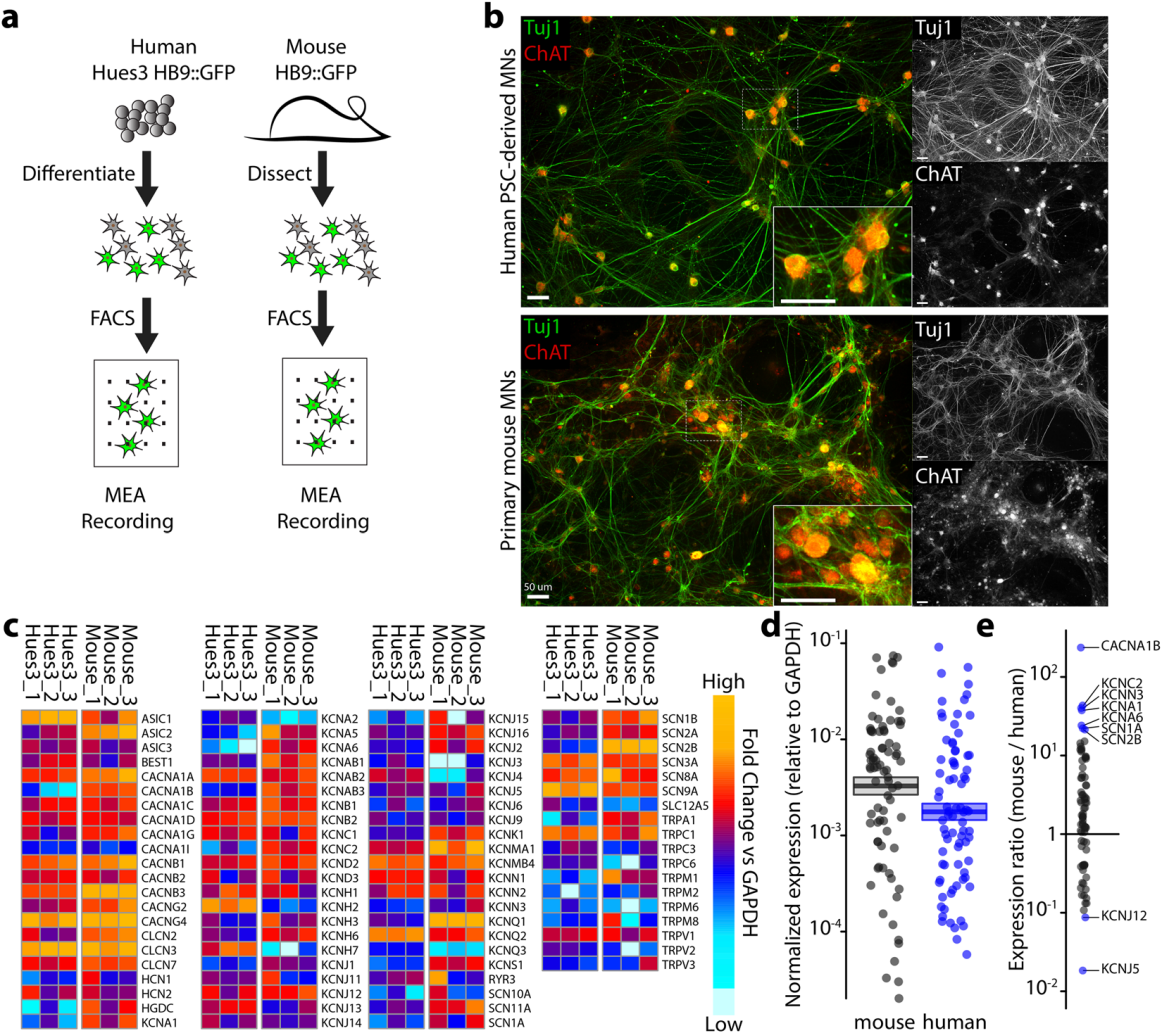
Molecular comparison of hPSC-derived and primary mouse motor neurons. (**a**) Strategies for purification of HUES3 HB9::GFP human motor neurons and Hb9::GFP primary mouse motor neurons. (**b**) Immunostaining of human (upper panel) and mouse (lower panel) motor neuron cultures 2 days following FACS purification. (**c**) Heat map of ion channel expression in human and mouse motor neurons in triplicate 2 days following FACS purification. Orange indicates high gene expression and cyan indicates low gene expression, normalized to GAPDH levels by batch. (**d**) Human PSC-derived (blue) and mouse primary (grey) motor neuron gene expression. Each circle indicates the median expression of an individual gene; line and shaded area represent mean and standard error. Mouse genes are skewed towards higher expression relative to human. (**e**) Relative gene expression between hPSC-derived and mouse primary motor neurons.

HB9 is an early marker of post-mitotic spinal motor neurons and is expressed in both hPSC-derived and embryonic mouse spinal motor neurons^16^. We leveraged this consistent selective expression to obtain highly congruent populations of mouse and human spinal motor neurons in parallel experimental paradigms (Fig. 1a). To derive human motor neuron-enriched cultures, a hPSC line with a GFP expression marker driven by the HB9 promoter was differentiated using a monolayer strategy, and HB9:GFP-positive neurons were isolated using fluorescence-activated cell sorting (FACS)^3,17^. To obtain mouse motor neuron-enriched cultures, we used a transgenic Hb9 (*Hlxb9*):GFP mouse strain, which matches endogenous Hb9 expression^18^, and FACS-purified motor neurons from embryonic mouse spinal cords (12.5–13.5 days post-conception). We chose this age based on the technical feasibility to generate a sufficiently large population of motor neurons and the established use of this protocol in the literature^19,20^. FACS gating parameters were similar for both hPSC-derived and mouse primary motor neurons (Supplementary Fig. 1). Two days after FACS purification, we confirmed that both human and mouse cultures maintained expression of spinal motor neuron markers βIII-Tubulin (Tuj1, Fig. 1b) and choline acetyltransferase (ChAT, Fig. 1b), an enzyme necessary for transmitter synthesis in cholinergic neurons and a typical marker of spinal motor neurons. We observed that 85.7 ± 4.5% (mean ± SEM, n = 3 independent sorts) of Tuj1-postive neurons expressed ChAT in hPSC-derived cultures, and 98.6 ± 5.8% (mean ± SEM, n = 4 independent sorts) of Tuj1-positive neurons expressed ChAT in mouse primary cultures.

To identify highly expressed ion channels on the RNA level, we used RT^2^ profiler PCR arrays to compare the expression of 84 neuronal ion channels (Fig. 1c). Separate arrays were used to quantify expression in human and mouse tissue to account for primer affinity differences. We broadly observed similar expression of voltage-gated calcium, potassium, sodium and calcium-activated potassium channels between human and mouse cells, although expression was higher overall in mouse cells relative to human cells (Fig. 1d). Some genes were more highly expressed in mouse neurons relative to human (e.g. CACNA1B, KCNC2, and SCN1A; Fig. 1e), whereas others were more highly expressed in human neurons (e.g. KCNJ5, KCNJ12; Fig. 1e). KCNQ (Kv7) channels were represented primarily by KCNQ2 in the human neurons.

### Characterization of spontaneous activity of hPSC-derived and mouse motor neurons

**Figure 2:**
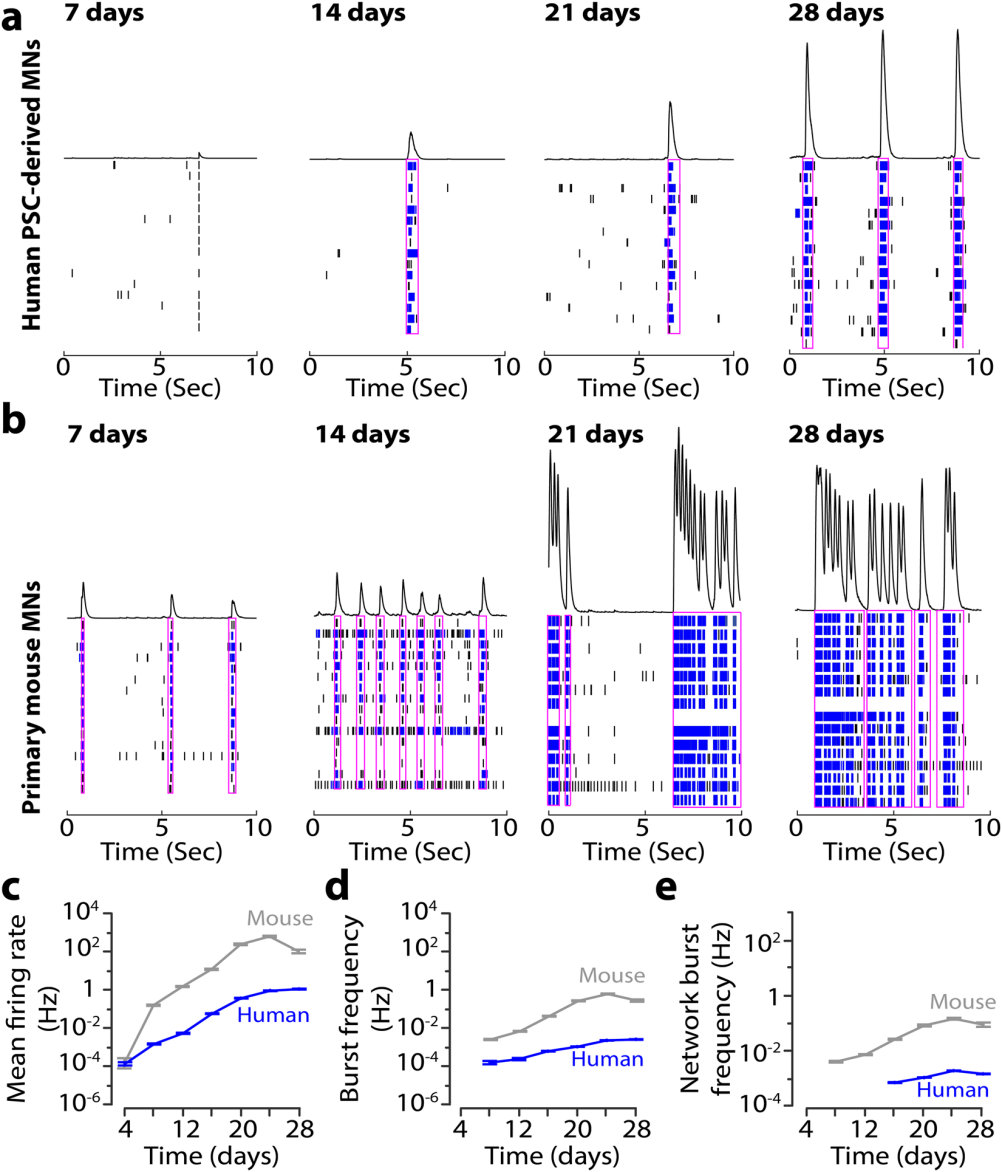
Firing and network bursts in hPSC-derived and primary mouse motor neurons. (**a**,**b**) Time course of activity development for hPSC-derived motor neurons (**a**) and primary mouse motor neurons (**b**) at 7, 14, 21, and 28 days after sorting. Raster plots show individual electrode spikes (black), within-electrode bursts (blue), and network bursts (pink rectangle). Traces above raster plots show population spike histogram. **c-e**, Longitudinal changes in mean firing rate (**c**), burst frequency (**d**), and network burst frequency (**e**) on log_10_ scale for purified hPSC-derived neurons (blue) and primary mouse motor neurons (grey).

To investigate the longitudinal firing and physiological properties of the human and mouse spinal motor neurons, we co-cultured hPSC-derived motor neurons or primary mouse motor neurons with primary mouse glia on MEAs, grids of extracellular micro-electrodes used to record action potentials (Fig. 2a,b). Glial cell co-culture facilitates the long-term adherence of neuronal cultures on the MEAs, and we chose neonatal mouse cortical glia based on prior experience obtaining functional co-cultures without contamination by cortical neurons in the glia^10^.

Human and mouse motor neurons demonstrated large differences in spontaneous firing activity over the four week recording period, although each showed a similar increase in spontaneous firing over four weeks (Fig. 2a,b). Spikes detected in the MEA recordings were classified into single neuronal spikes, bursts containing repeated spikes recorded on the same electrode, and network bursts across multiple electrodes in the same well. Although hPSC-derived motor neuron cultures consistently displayed less activity compared with mouse motor neuron cultures, by three weeks after sorting, hPSC-derived motor neurons displayed robust burst firing and network bursts (Fig. 2a,b). Mean firing rate (MFR) and burst frequency (BF, bursting within an individual electrode) both peaked at 25–28 days after sorting for hPSC-derived motor neurons (MFR: 1.68 Hz ± 0.026; BF: 0.07 Hz ± 0.001; Fig. 2c,d, Supplementary Table 1) and at days 21–24 for the mouse motor neurons (MFR: 27.92 Hz ± 0.46; BF: 0.94 Hz ± 0.013; Fig. 2c,d, Supplementary Table 1). Network burst frequency (NBF, synchronization among electrodes) peaked at days 21–24 after sorting for both hPSC-derived and mouse motor neurons (hPSC NBF: 0.036 Hz ± 0.0005; mouse NBF: 0.83 Hz ± 0.01; Fig. 2e, Supplementary Table 1). Based on these results, we chose to assess network function and intrinsic excitability at three weeks in culture, at the peak of network activity for both cultures.

### Pharmacology of networks

**Figure 3:**
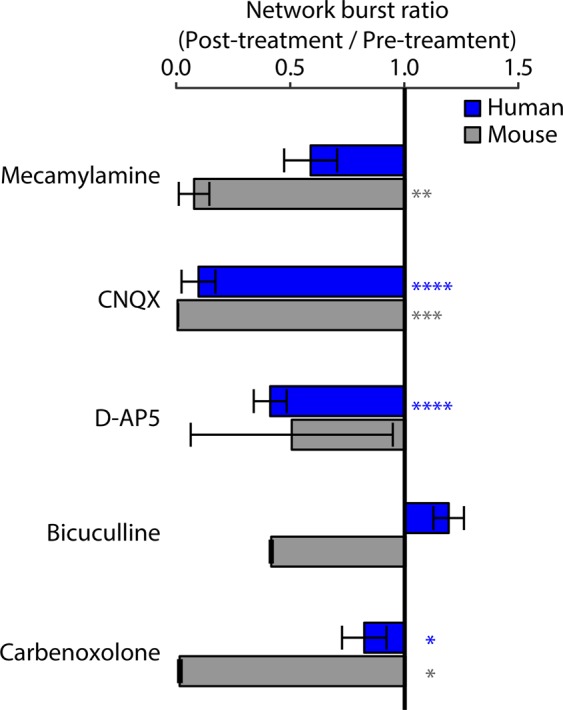
Identification of transmitters driving network bursts. Effect of specific antagonists on network bursts in FACS-purified hPSC-derived motor neurons (blue) and mouse motor neurons (grey). Values represent ratio of network burst frequency after treatment relative to network burst frequency before treatment. Bars and error bars represent mean and standard error. P-values are represented as stars with the following notation: ≤0.0001 (****), ≤0.001(***), ≤0.01(**), ≤0.05 (*) and >0.05 (-).

The formation of neural networks requires neuronal specification in terms of the synthesis and synaptic release of individual or multiple distinct neurotransmitters^21^. To determine which neurotransmitters drove network activity in the motor neuron cultures, we applied blockers of common neurotransmitters once network bursts were well established, after at least three weeks in culture (Fig. 3, Supplementary Fig. 2). Drug effects were measured relative to vehicle treatment independently for hPSC-derived and mouse motor neurons.

Human and mouse motor neurons express ChAT, and acetylcholine is the primary transmitter at the neuromuscular junction *in vivo*; therefore, we anticipated that networks would be driven largely by acetylcholine. Mouse motor neurons showed robust inhibition of network bursts following treatment with the broad nicotinic blocker mecamylamine^22^. However, we observed only a modest reduction of network bursts in cultures of human motor neurons, leading us to consider alternative neurotransmitter systems. Surprisingly, the ionotropic glutamate receptor antagonist 6-cyano-7-nitroquinoxaline-2,3-dione (CNQX), which blocks the α-amino-3-hydroxy-5-methyl-4-isoxazolepropionic acid (AMPA) subset of glutamate receptors, nearly eliminated the synchronized bursts in both human and mouse motor neurons. D-(-)-2-Amino-5-phosphonopentanoic acid (D-AP5), which blocks the N-methyl-D-aspartate (NMDA) subset of glutamate receptors, yielded a partial inhibition in both species, although the effect was highly variable in mouse cultures. To assess for contamination from interneurons, we applied bicuculline, which blocks gamma-aminobutyric acid (GABA)-A receptors and did not observe a significant effect on network bursting in either species. Finally, we looked for direct electrical coupling between motor neurons using the gap junction inhibitor carbenoxolone and found that it reduced networking, consistent with the role of gap junctions in motor neuron development^23,24^. This effect was larger in mouse cultures compared with human cultures.

Because we did not expect glutamate blockers to exert such robust effects on motor neuron firing, we considered the possibility that the network activity resulted from contamination of motor neuron cultures by other cell types. Although the lack of response to bicuculline suggested no major contribution from GABA-ergic inhibitory interneurons, excitatory HB9-positive interneurons could have remained in the FACS purification and confounded our results^25^. To address this potential explanation, instead of the HB9::GFP hPSC line, we used an Islet::tdTomato hPSC line to purify motor neurons. We found similar network properties, including a strong sensitivity to glutamatergic blockers (Supplementary Fig. 3), consistent with our original findings using the HB9 reporter line.

We next investigated whether the network bursts and ability of CNQX to block them resulted from co-culture with primary mouse glia, which was critical for long-term adherence on the MEA plates. Culturing the neurons without glia led to a markedly reduced degree of firing, consistent with the facilitatory role of glia in neuronal maturation^26^; however, the synchronization of neuronal firing within wells still occurred in the absence of glia (Supplementary Fig. 4).

### Pharmacological profiles of motor neurons in desynchronized culture

**Figure 4:**
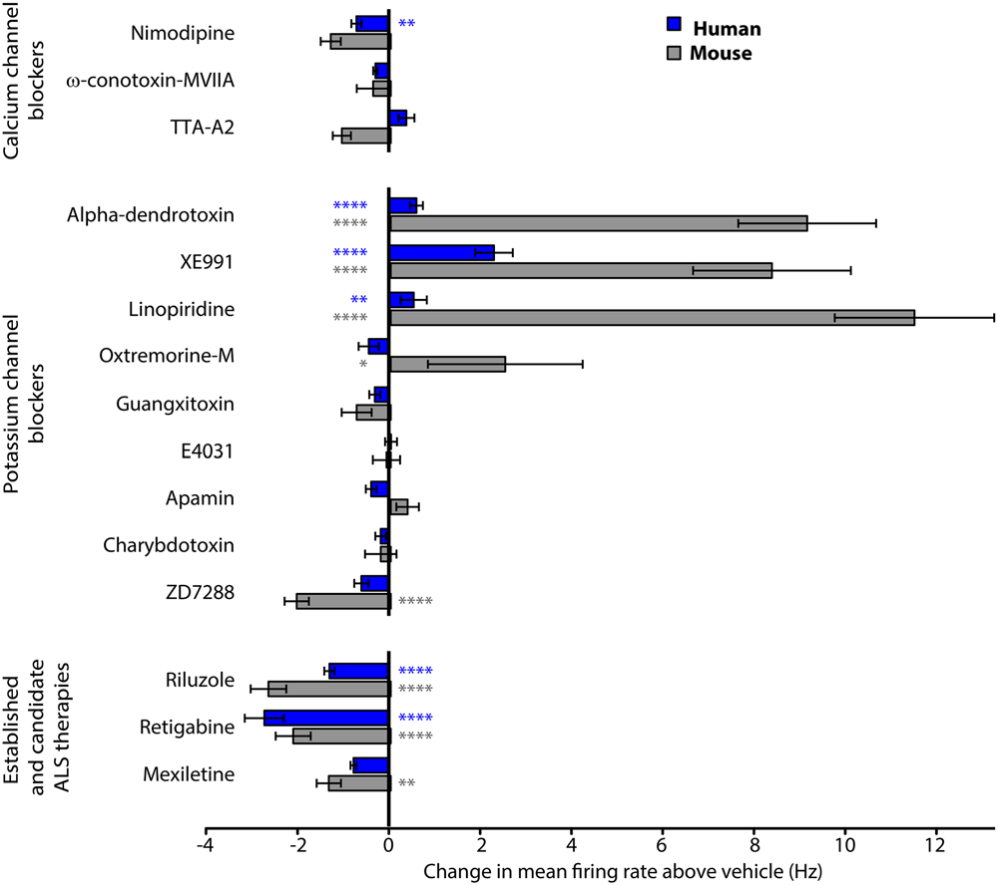
Pharmacological profile of human and mouse motor neurons after network burst block. The effects of drugs on mean firing rate in FACS-purified hPSC-derived (blue) and primary mouse (grey) motor neurons. Values show effects of individual drugs after subtracting the effect of vehicle. Bars and error bars show mean and standard error. P-values are represented as stars with the following notation: ≤0.0001 (****), ≤0.001(***), ≤0.01(**), ≤0.05 (*), and >0.05 (-).

We then wanted to ascertain which channels contributed to the spontaneous firing of the motor neurons in the absence of network activity. We blocked synaptic activity using a cocktail of CNQX and D-AP5 and took advantage of the specificity of a host of different toxins and drugs to assess the roles of a broad range of ion channels in determining intrinsic excitability. In choosing the drugs to evaluate, we considered both the expression of target channels in our motor neuron cultures as well the documented required concentrations from the literature (Supplementary Table 2). We examined the effects of the drugs on mean firing rate for both hPSC-derived and mouse purified spinal motor neurons (Fig. 4). Drug effects were compared to vehicle treatment using mixed models independently for hPSC-derived and mouse motor neuron cultures. Voltage-gated calcium channels play major roles in controlling neuronal firing and excitability and we used nimodipine^27^, ω-conotoxin-MVIIA^28^, and TTA-A2^29^ to assess the contribution of L-type (Ca_V_1), N-type (Ca_V_2.2) and T-type (Ca_V_3) calcium channels, respectively^30^. Effects of all three drugs were modest for both mouse and human neurons, with only the effect of nimodipine on hPSC-derived motor neurons reaching significance (−0.716 Hz ± 0.11; p ≤ 0.01; Fig. 4).

KCNQ (Kv7) potassium channel openers have previously been shown to reduce motor neuron activity in both hPSC-derived and mouse motor neurons^10,31^; however, how other potassium channels regulate hPSC motor neuron firing and whether those same channels control primary mouse motor neuron firing has not been evaluated. Both hPSC-derived and mouse motor neurons showed an increased mean firing rate after application of the Kv1 blocker α-dendrotoxin (hPSC-derived: 0.69 Hz ± 0.14; p ≤ 0.0001; mouse: 9.12 Hz ± 1.51; p ≤ 0.0001; Fig. 4)^32^. Consistent with prior studies on Kv7 channels, both hPSC and primary mouse motor neurons displayed increased mean firing rate following the application of the Kv7 blockers XE991^33^ and linopiridine^33^ (hPSC-derived: XE991 2.31 Hz ± 0.41; p ≤ 0.0001; linopiridine 0.54 Hz ± 0.28; p ≤ 0.01; Fig. 4; mouse: XE991 8.35 Hz ± 1.72; p ≤ 0.0001; linopiridine 11.47 Hz ± 1.74; p ≤ 0.0001; Fig. 4). Notably, mouse motor neurons displayed a strong increase in mean firing rate in response to the muscarinic agonist oxotremorine-M^33^, which blocks Kv7 channels through metabotropic modulation of Gq^33^ (2.50 Hz ± 1.69; p ≤ 0.05; Fig. 4), while hPSC-motor neurons lacked this response. Neither culture type responded to Kv2, Kv11, SK, or BK channel blockers (guanxitoxin^34^, E-4301^35^, apamin^36^, and charybdotoxin^36^, respectively).

Hyperpolarization-activated cyclic nucleotide-gated HCN channels, which are members of the voltage-gated potassium channel family but function primarily to conduct sodium inwards, play major roles in neuronal rhythmicity^37,38^. The HCN channel blocker ZD7288^39^ showed a trend toward reduction of firing in hPSC motor neurons and a significant decrease in mouse motor neurons (−2.06 Hz ± 0.26; p ≤ 0.0001; Fig. 4).

Because hPSC-based neuronal models are used to identify, assess and compare potential therapeutics, we evaluated the responses of both hPSC-derived and mouse motor neurons to riluzole, the long-standing ALS approved therapeutic, as well as retigabine and mexiletine, which are both under evaluation in clinical trials^10,40,41^. Both riluzole, which reduces glutamate release from astrocytes and blocks persistent sodium currents in neurons^42^, and retigabine, a KCNQ channel opener^43^, decreased the mean firing rates of hPSC-derived (Riluzole: −1.31 Hz ± 0.11; p ≤ 0.0001; Retigabine −2.74 Hz ± 0.43; p ≤ 0.0001; Fig. 4) and mouse motor neurons (Riluzole: −2.68 Hz ± 0.39; p ≤ 0.0001; Retigabine −2.13 Hz ± 0.38; p ≤ 0.0001; Fig. 4). However, the sodium channel blocker mexiletine significantly decreased the mean firing rate only for mouse motor neurons (−1.35 Hz ± 0.26; p ≤ 0.01; Fig. 4)^44^.

## Discussion

The ability to generate pluripotent stem cells from individual patient samples holds great potential for improving mechanistic understanding of pathogenesis, developing therapeutics targeted to defined cellular and patient subsets, and replacing populations of cells affected in specific diseases^1^. Current differentiation techniques have produced a broad range of specific disease-relevant neuronal subtypes; however, the confirmation of cellular identity has been largely on the RNA and protein levels^45^, with physiological function limited to a coarse assessment of firing ability and gross measurements of sodium and potassium channels.

Because of the key relevance of physiology to neuronal function, we thought it important to demonstrate that the functional properties of hPSC-derived neurons approximate those of *bona fide* primary neurons. In particular, because hPSC-derived motor neurons are being used to identify candidate therapeutics which modify neuronal excitability, we wanted to know whether specific drugs in practice and under evaluation yielded similar effects on hPSC-derived and primary motor neurons. Using established techniques for differentiating spinal motor neurons from hPSCs and purifying both hPSC-derived and primary mouse spinal motor neurons using the same reporter, we were well positioned to ask these questions. The results of our study are reassuring and support the relevance of hPSC-derived spinal motor neurons as physiologically-competent models for primary motor neurons.

Caveats to our study include the immaturity of the mouse primary motor neurons used as a “gold standard”, although the ability to isolate and FACS-purify motor neurons from older animals is technically limiting^19^. Furthermore, prior RNA-sequencing studies have shown that hPSC-derived motor neurons more closely resemble embryonic than adult neurons, thus justifying the timing of our comparison^6^. We evaluated hPSC-derived motor neurons at a time of robust firing; nonetheless, more time in culture may produce greater cellular maturation, which may be required for muscarinic modulators to complete the pathway to M-channel gating.

Our study has largely been limited to two hPSC-lines and a single mouse strain. Thus, potential exists for markedly different physiological behaviour of motor neurons from different hPSC lines or mouse variants. Because we are unable to use human primary motor neurons, we cannot distinguish differences resulting from primary human versus primary mouse identity. Although we considered the additional comparison of mouse stem cell-derived motor neurons, we thought the primary added value of the study came from the hPSC to primary mouse comparison and as such focused on that.

We found the profound glutamatergic networking of the HB9-purified hPSC-derived motor neurons to be surprising, despite a similar result in unsorted primary rat motor neurons^46^. Nonetheless, consistent findings in the purified primary mouse neurons were reassuring. There is a strong precedent for multiple transmitter types within the same neurons^47^. Furthermore, glutamatergic release from spinal motor neurons has been documented in physiological studies of mouse and rat motor neurons^48,49^, although the extent and functional importance of this capacity have not been thoroughly established.

The limited cholinergic contribution to the hPSC motor neuron network – despite the robust ChAT signal observed by immunohistochemistry – was also unexpected. Indeed, this result emphasizes the critical importance of using functional physiological criteria in characterizing neurons. One possible explanation is that cholinergic functional maturation in the hPSC motor neurons has a greater requirement for support from neuromuscular junctions, which normally provide the proper synaptic and signalling context^50^. Such an interpretation would be consistent with prior findings that central synaptic output from motor neurons is largely glutamatergic^49^, as opposed to cholinergic in the periphery. While neurons can de-differentiate in culture^51^, we did not observe such an effect in the mouse primary motor neurons.

Although we demonstrated via immunohistochemistry and pharmacology the overwhelming enrichment of motor neurons in both mouse and hPSC-derived cultures, we cannot definitively exclude the possibility of contamination from a subset of non-motor neurons which significantly drive network activity, particularly given the known population of Hb9-positive excitatory interneurons^52^. However, the similarity of the findings when sorting based on both Hb9 and Islet argue against this possibility.

The role of glia in promoting neuronal maturation has been well documented^26^. While we considered comparing mouse neurons co-cultured with mouse glia to human neurons co-cultured with human glia, the primary goal of the study was to assess differences between mouse and human neurons. Thus, we thought it best to address this question using the same glia source. Pairing by species would introduce an additional confounder in interpreting the differences between mouse and human neurons. While we cannot rule out effects of glia on the individual channel families determining neuronal excitability, we confirmed that the network bursts were present in motor neurons alone (Supplementary Fig. 4) and thus did not result from glia co-culture.

Stem cell-based models that capture the molecular and functional identity of their *bone fide* primary cell types may be better poised to recapitulate disease pathology. This is particularly the case for neurological diseases, in which compromised physiological function defines disease progression. The results here, showing that hPSC-derived motor neurons capture quintessential physiological features of their primary mouse counterparts, provide reassurance for the broad and ongoing use of hPSC-derived motor neurons in human disease modelling.

## Methods

All experiments were conducted under protocols approved by the Massachusetts General Hospital Institutional Review Board, Institutional Animal Care and Use Committee, and Partners Institutional Biosafety Committee. All methods were performed in accordance with relevant guidelines and regulations.

### Maintenance and culture of human pluripotent stem cells

The HUES3 HB9::GFP line^17^ (kindly provided by K. Eggan) and the WA09 (H9) Isl::tdTomato (kindly provided by J. Ichida) were maintained in mTeSR (Stem Cell Technologies, 05850) on Matrigel (Corning, 354277) until 90% confluent, when they were either passaged or differentiated.

### 2D Differentiation of hPSCs into motor neurons

Differentiation of hPSCs into motor neurons was performed as previously published^3^. Cells were fed daily for a period of 14 days with NB/B27: a 1:1 mixture of Neurobasal (Life Technologies, 21103049) and DMEM/F12 (Life Technologies, 11320-082) supplemented with GlutaMAX (Life Technologies, 35050061), NEAA (Corning, 25-025-CI), Pen/Strep (Life Technologies, 15070-063), N2 (Life Technologies, 17502048), and B27 (Life Technologies, 17504044). From day 0 to day five the media was supplemented with dual SMAD inhibitors SB-431542 (10 µM, DNSK, DNSK-KI-12) and LDN-193189 (100 nM, Stemgent, 04-0074-02) as well as Retinoic acid (1 µM, Sigma, R2625) and SAG (1 µM, DNSK, DNSK-SMO-1). From day six through day 13, the dual SMAD inhibitors were replaced with DAPT (5 µM, DNSK, DNSK-EI-01) and SU-5402 (5 µM, DNSK, DNSK-KI-11). Dissociation of the 2D motor neuron culture was performed on day 14 by incubating the culture in StemPro Accutase Cell Dissociation Reagent (Thermo Fisher Scientific, A1110501) with DNase (Worthington, LK003172) for one hour at 37 °C. The resulting cell suspension was dissociated mechanically by pipetting 20 times followed by centrifugation at 200 rcf for six minutes. The cell pellet was resuspended in sterile filtered sort buffer consisting of 1% BSA (Sigma, A9576), 15 mM HEPES (Teknova, H1030), 1% Pen/Strep (Life Technologies, 15070-063), 2 mM EDTA (Fisher Scientific, BP2482) in PBS (Life Technologies, 10010049) with DAPI (Thermo Fisher Scientific, D1306) and filtered through a 40 µm nylon mesh (Fisher Scientific, 22363547) immediately prior to FACS sorting.

### FACS sorting and long-term culture of hPSC derived neurons

FACS sorting was performed with either a BD FACSAria II or a BD FACSAria Fusion. After sorting, motor neurons were maintained in motor neuron maintenance media consisting of Neurobasal media (Life Technologies, 21103049), GlutaMAX (Life Technologies, 35050061), NEAA (Corning, 25-025-CI), N2 (Life Technologies, 17502048), B27 (Life Technologies, 17504044), and Pen/Strep (Life Technologies, 15070-063) supplemented with GDNF (10 ng/ml, Life Technologies, PHC7044), BDNF (10 ng/ml, Life Technologies, PHC7074), and CNTF (10 ng/ml, Life Technologies, PHC7015) with half media changes 3 times per week.

### Primary mouse motor neuron dissection and maintenance

Embryonic motor neurons were harvested from E12.5–13.5 spinal cords of B6.Cg-Tg(Hlxb9-GFP)1Tmj/J (Hb9:GFP) mice (Jackson Laboratory, strain 005029) under the dissecting microscope as previously described^20^. After spinal cord isolation, the tissue was submerged in PBS (Life Technologies, 10010049) at 4 °C followed by incubation with Trypsin-EDTA (Life Technologies, 25200-056) at 37 °C for 10 minutes with agitation. The Trypsin-EDTA mixture was then carefully replaced with 1 ml of NB/B27 with DNase (Worthington, LK003172) and 0.4% BSA (Sigma, A9576), and the tissue was mechanically dissociated by pipetting 20 times with a fire-polished Pasteur pipette. The resulting cell suspension was filtered through a 100 µm cell strainer (Fisher Scientific, 22362549), centrifuged at 150 rcf for five minutes and resuspended in the same sort buffer composition as for the hPSC derived motor neurons for FACS sorting.

### Primary glial isolation and maintenance

The cerebral cortex from mouse pups ranging P1-P3 was dissected and placed into HBSS (Life Technologies, 14185052) buffer containing 1% Pen/Strep (Life Technologies, 15070-063) and 10 mM HEPES (Teknova, H1030). The tissue was then dissociated enzymatically with 0.5 ml of 0.25% Trypsin-EDTA (Life Technologies, 25200-056) for 5–8 minutes at 37 °C. The trypsin was then inactivated by adding 1.5 ml of glial media: MEM (Life Technologies, 10370) with 10% heat inactivated donor equine serum (Hyclone, SH30074.03HI), 3% Glucose (Teknova, 76061-922), and 1% Pen/Strep (Life Technologies, 15070-063). The resulting suspension was mechanically dissociated and then centrifuged at 200 rcf for nine mins, resuspended in glial media and slowly filtered using a 100 µm mesh filter, after which the filtrate was then seeded in tissue culture vessels coated with Poly-D-Lysine (PDL) (Sigma, P6407).

### Immunocytochemistry

Cells were washed for 5 minutes in PBS and then fixed with 4% (w/v) paraformaldehyde (Thermo Fisher Scientific, 28908) for 15 minutes, followed by three washes, 5 minutes each, in PbTr (PBS with 0.125% Triton-X 100; Millipore Sigma, 9400). Following an additional 10 minutes permeabilization with PbTr, the cells were blocked in PbTr with 5% normal donkey serum (Abcam, ab7475) for 45 minutes. Primary antibodies (ChAT, 1:100, EMD Milipore AB144P; Tuj1, 1:1000, BioLegend 801202) were diluted in blocking buffer and cells were incubated overnight at 4 °C in a humidified chamber. After three 5 minute washes with PbTr, cells were incubated in the dark for an hour with secondary antibodies (Alexa Fluor secondary antibodies, ThermoFisher Scientific, at a 1:1000 dilution). Cells were finally washed three times with PbTr, and the slides were mounted in Prolong Diamond with DAPI (Life Technologies, P36962). Slides were mounted overnight at room temperature prior to imaging.

### RT^2^ profiler PCR arrays

Gene expression analysis of FACS sorted Hb9::GFP mouse motor neurons and HUES3 HB9::GFP hPSC-derived motor neurons was performed by qPCR array the RT^2^ Profiler PCR Array Mouse Neuronal Ion Channels (Qiagen, 330231 PAMM-036Z) and RT^2^ Profiler PCR Array Human Neuronal Ion Channels (Qiagen, 330231 PAHS-036Z), respectively. RNA was extracted of sorted neurons immediately after FACS using Trizol and 2 µg of RNA were converted to cDNA with the High-Capacity cDNA Reverse Transcription Kit (Thermo Fisher Scientific, 4368814). qPCR was performed with iQ SYBR Green Supermix (Biorad, 1708882) in a Biorad CFX96 according to the array manufacturer’s protocol.

### MEA motor neuron cultures

48-well MEA plates (Axion Biosystems) were coated with 50 µg/ml PDL in 5 µl drops in the centers of the wells at room temperature for 1 h. After three washes with PBS, the plates were coated with laminin (Life Technologies, 23017-015) for 1 hour. Sorted motor neurons (20,000, with or without 1:1 mouse primary glia) were plated in a volume of 5 µl per well and allowed to attach for 1 hour at 37 °C. Motor neuron maintenance media (see above) was added to each well to a total volume of 300 µL. Longitudinal MEA recordings were performed immediately prior to media changes three times per week by loading the MEA plate into the pre-warmed Maestro MEA plate reader (Axion Biosystems). After allowing for five minutes of equilibration, the plate was recorded for five minutes.

Drugs were pre-mixed at 10x concentration in 48 well plates (see Supplemental Table 2 for concentrations). After the MEA plate was equilibrated, a five-minute baseline recording was taken. For network pharmacology experiments, one tenth of the final well volume carrying each drug was then added to the appropriate wells using a multichannel pipette, angling the tips to the side of the wells just below the meniscus. A post-drug recording was taken thirty minutes after drug addition. For desynchronized experiments, a cocktail of CNQX and D-AP5 were first similarly added to the cultures and were allowed an hour to block network activity. A five-minute post-block baseline recording was taken before the main drugs were added as described above, with the final recordings recorded 30 minutes after drug addition.

### Statistical analysis

For network burst analysis (Fig. 3), we used two time points, baseline and 30 minutes after drug addition, with five minute recordings at each point. We cleaned the data by removing wells that were not recorded at baseline and calculated ratios of number of network bursts at 30 minutes compared to that at baseline. Network bursts were identified using Axion Axis software (Version 2.1). Because the data did not follow a normal distribution, one-sided Wilcoxon sign test with Bonferroni correction was used to test the null hypothesis that the median of the ratio distribution for each drug is equal to that of control for hPSCs and mouse motor neuron separately.

For drug effects in blocked neurons (Fig. 4), we first cleaned the data by removing any electrodes that were not recorded at baseline and those with mean firing rate greater than 10 or less than 0.01 Hz. We utilized a mixed effect model with interaction of two fixed effects, time and drug, and a random intercept to analyse the mean firing rate for each species across different drugs. Through model simulation, we selected two models with the lowest Akaike Information criterion (AIC) for hPSCs and mouse motor neurons. We used emmeans (estimated marginal means) in R (version 3.4.2) to predict the mean firing rate and performed one-sided test with false discovery rate correction across the drugs for each species with a null hypothesis that linear mean firing rate change of each drug was equal to 0.

## Supplementary information

Supplementary Info

## Data Availability

No genomic datasets were generated or analysed during the current study.

## References

[1] Saha K, Jaenisch R (2009). Technical challenges in using human induced pluripotent stem cells to model disease. Cell Stem Cell.

[2] Gibbs RM (2018). Toward Precision Medicine for Neurological and Neuropsychiatric Disorders. Cell Stem Cell.

[3] Klim JR (2019). ALS-implicated protein TDP-43 sustains levels of STMN2, a mediator of motor neuron growth and repair. Nat Neurosci.

[4] Pasinelli P, Brown RH (2006). Molecular biology of amyotrophic lateral sclerosis: insights from genetics. Nat Rev Neurosci.

[5] Dimos JT (2008). Induced pluripotent stem cells generated from patients with ALS can be differentiated into motor neurons. Science.

[6] Ho R (2016). ALS disrupts spinal motor neuron maturation and aging pathways within gene co-expression networks. Nat Neurosci.

[7] Mann M, Jensen ON (2003). Proteomic analysis of post-translational modifications. Nat Biotechnol.

[8] Bean BP (2007). The action potential in mammalian central neurons. Nat Rev Neurosci.

[9] Kullmann DM, Waxman SG (2010). Neurological channelopathies: new insights into disease mechanisms and ion channel function. J Physiol (Lond).

[10] Wainger BJ (2014). Intrinsic membrane hyperexcitability of amyotrophic lateral sclerosis patient-derived motor neurons. CellReports.

[11] Bae JS, Simon NG, Menon P, Vucic S, Kiernan MC (2013). The puzzling case of hyperexcitability in amyotrophic lateral sclerosis. J Clin Neurol.

[12] Devlin A-C (2015). Human iPSC-derived motoneurons harbouring TARDBP or C9ORF72 ALS mutations are dysfunctional despite maintaining viability. Nat Commun.

[13] Chan CS (2007). ‘Rejuvenation’ protects neurons in mouse models of Parkinson’s disease. Nature.

[14] Busche MA (2008). Clusters of hyperactive neurons near amyloid plaques in a mouse model of Alzheimer’s disease. Science.

[15] Amatniek JC (2006). Incidence and predictors of seizures in patients with Alzheimer’s disease. Epilepsia.

[16] Kanning KC, Kaplan A, Henderson CE (2010). Motor neuron diversity in development and disease. Annu Rev Neurosci.

[17] Boulting GL (2011). A functionally characterized test set of human induced pluripotent stem cells. Nat Biotechnol.

[18] Wichterle H, Lieberam I, Porter JA, Jessell TM (2002). Directed differentiation of embryonic stem cells into motor neurons. Cell.

[19] Wiese S (2010). Isolation and enrichment of embryonic mouse motoneurons from the lumbar spinal cord of individual mouse embryos. Nat Protoc.

[20] Son EY (2011). Conversion of mouse and human fibroblasts into functional spinal motor neurons. Cell Stem Cell.

[21] Zhang LI, Poo MM (2001). Electrical activity and development of neural circuits. Nat Neurosci.

[22] Nishimaru H, Restrepo CE, Kiehn O (2006). Activity of Renshaw cells during locomotor-like rhythmic activity in the isolated spinal cord of neonatal mice. J Neurosci.

[23] Montague K (2017). The assembly of developing motor neurons depends on an interplay between spontaneous activity, type II cadherins and gap junctions. Development.

[24] Alvarez-Maubecin V, Garcia-Hernandez F, Williams JT, Van Bockstaele EJ (2000). Functional coupling between neurons and glia. Journal of Neuroscience.

[25] Ziskind-Conhaim L, Mentis GZ, Wiesner EP, Titus DJ (2010). Synaptic integration of rhythmogenic neurons in the locomotor circuitry: the case of Hb9 interneurons. Ann N Y Acad Sci.

[26] Johnson MA, Weick JP, Pearce RA, Zhang S-C (2007). Functional neural development from human embryonic stem cells: accelerated synaptic activity via astrocyte coculture. J Neurosci.

[27] Randall A, Tsien RW (1995). Pharmacological dissection of multiple types of Ca2+ channel currents in rat cerebellar granule neurons. J Neurosci.

[28] Stocker JW, Nadasdi L, Aldrich RW, Tsien RW (1997). Preferential interaction of omega-conotoxins with inactivated N-type Ca2+ channels. J Neurosci.

[29] Kraus RL (2010). *In vitro* characterization of T-type calcium channel antagonist TTA-A2 and *in vivo* effects on arousal in mice. Journal of Pharmacology and Experimental Therapeutics.

[30] Simms BA, Zamponi GW (2014). Neuronal Voltage-Gated Calcium Channels: Structure, Function, and Dysfunction. Neuron.

[31] Lombardo J, Harrington MA (2016). Nonreciprocal mechanisms in up- and downregulation of spinal motoneuron excitability by modulators of KCNQ/Kv7 channels. J Neurophysiol.

[32] Grissmer S (1994). Pharmacological characterization of five cloned voltage-gated K+ channels, types Kv1.1, 1.2, 1.3, 1.5, and 3.1, stably expressed in mammalian cell lines. Mol Pharmacol.

[33] Brown DA, Passmore GM (2009). Neural KCNQ (Kv7) channels. Br J Pharmacol.

[34] Liu PW, Bean BP (2014). Kv2 Channel Regulation of Action Potential Repolarization and Firing Patterns in Superior Cervical Ganglion Neurons and Hippocampal CA1 Pyramidal Neurons. Journal of Neuroscience.

[35] Meves H, Schwarz JR, Wulfsen I (1999). Separation of M-like current and ERG current in NG108-15 cells. Br J Pharmacol.

[36] Vergara C, Latorre R, Marrion NV, Adelman JP (1998). Calcium-activated potassium channels. Curr Opin Neurobiol.

[37] Wainger BJ, DeGennaro M, Santoro B, Siegelbaum SA, Tibbs GR (2001). Molecular mechanism of cAMP modulation of HCN pacemaker channels. Nature.

[38] Postea Otilia, Biel Martin (2011). Exploring HCN channels as novel drug targets. Nature Reviews Drug Discovery.

[39] Robinson RB, Siegelbaum SA (2003). Hyperpolarization-activated cation currents: from molecules to physiological function. Annu Rev Physiol.

[40] Lacomblez L, Bensimon G, Leigh PN, Guillet P, Meininger V (1996). Dose-ranging study of riluzole in amyotrophic lateral sclerosis. Amyotrophic Lateral Sclerosis/Riluzole Study Group II. Lancet.

[41] Weiss MD (2016). A randomized trial of mexiletine in ALS: Safety and effects on muscle cramps and progression. Neurology.

[42] Doble A (1996). The pharmacology and mechanism of action of riluzole. Neurology.

[43] Gunthorpe MJ, Large CH, Sankar R (2012). The mechanism of action of retigabine (ezogabine), a first-in-class K+ channel opener for the treatment of epilepsy. Epilepsia.

[44] Catterall WA, Goldin AL, Waxman SG (2005). International Union of Pharmacology. XLVII. Nomenclature and structure-function relationships of voltage-gated sodium channels. Pharmacol Rev.

[45] Sances S (2016). Modeling ALS with motor neurons derived from human induced pluripotent stem cells. Nat Neurosci.

[46] Zhang H-M, Robinson N, Gómez-Curet I, Wang W, Harrington MA (2009). Neuronal and network activity in networks of cultured spinal motor neurons. Neuroreport.

[47] Ren J (2011). Habenula ‘cholinergic’ neurons co-release glutamate and acetylcholine and activate postsynaptic neurons via distinct transmission modes. Neuron.

[48] Nishimaru H, Restrepo CE, Ryge J, Yanagawa Y, Kiehn O (2005). Mammalian motor neurons corelease glutamate and acetylcholine at central synapses. Proc Natl Acad Sci USA.

[49] Zhang H, Wu C-Y, Wang W, Harrington MA (2011). Interneuronal synapses formed by motor neurons appear to be glutamatergic. Neuroreport.

[50] Martinou JC, Martinou I, Kato AC (1992). Cholinergic differentiation factor (CDF/LIF) promotes survival of isolated rat embryonic motoneurons *in vitro*. Neuron.

[51] Gage FH (2000). Mammalian neural stem cells. Science.

[52] Ziskind-Conhaim, L., Wu, L. & Wiesner, E. P. Persistent sodium current contributes to induced voltage oscillations in locomotor-related hb9 interneurons in the mouse spinal cord. *J Neurophysiol* **100**, 2254–2264 (2008). 10.1152/jn.90437.2008 PMC2576213 18667543

