# Supplementary material for: Pharmacological Profiling of Purified Human Stem Cell-Derived and Primary Mouse Motor Neurons
Source: Sci Rep. 2019 Jul 25;9:10835. doi: 10.1038/s41598-019-47203-7 (PMC6658502; doi:10.1038/s41598-019-47203-7)
Supplement: Supplementary file 1 — Supplementary Info [file 41598_2019_47203_MOESM1_ESM.pdf]

Supplementary Information

Pharmacological Profiling of Purified Human Stem Cell-Derived and Primary Mouse Motor Neurons

Daniel Moakley[1,5], Joan Koh[1,5], Joao D. Pereira[1], Daniel M. DuBreuil[1], Anna-Claire Devlin[1], Kevin Zhu[1], Eugene Berezovski[1] & Brian J. Wainger[1-4, *]

[1]Department of Neurology, Massachusetts General Hospital, Harvard Medical School, Boston, MA 02114, USA

[2]Department of Anesthesiology, Critical Care and Pain Medicine, Massachusetts General Hospital, Boston MA 02114, USA

[3]Harvard Stem Cell Institute, Cambridge MA 02138, USA

[4]Broad Institute of Harvard University and MIT, Cambridge MA 02142, USA

[5]These authors contributed equally to this work.

[*]Correspondence should be addressed to B.J.W (brian.wainger@mgh.harvard.edu).

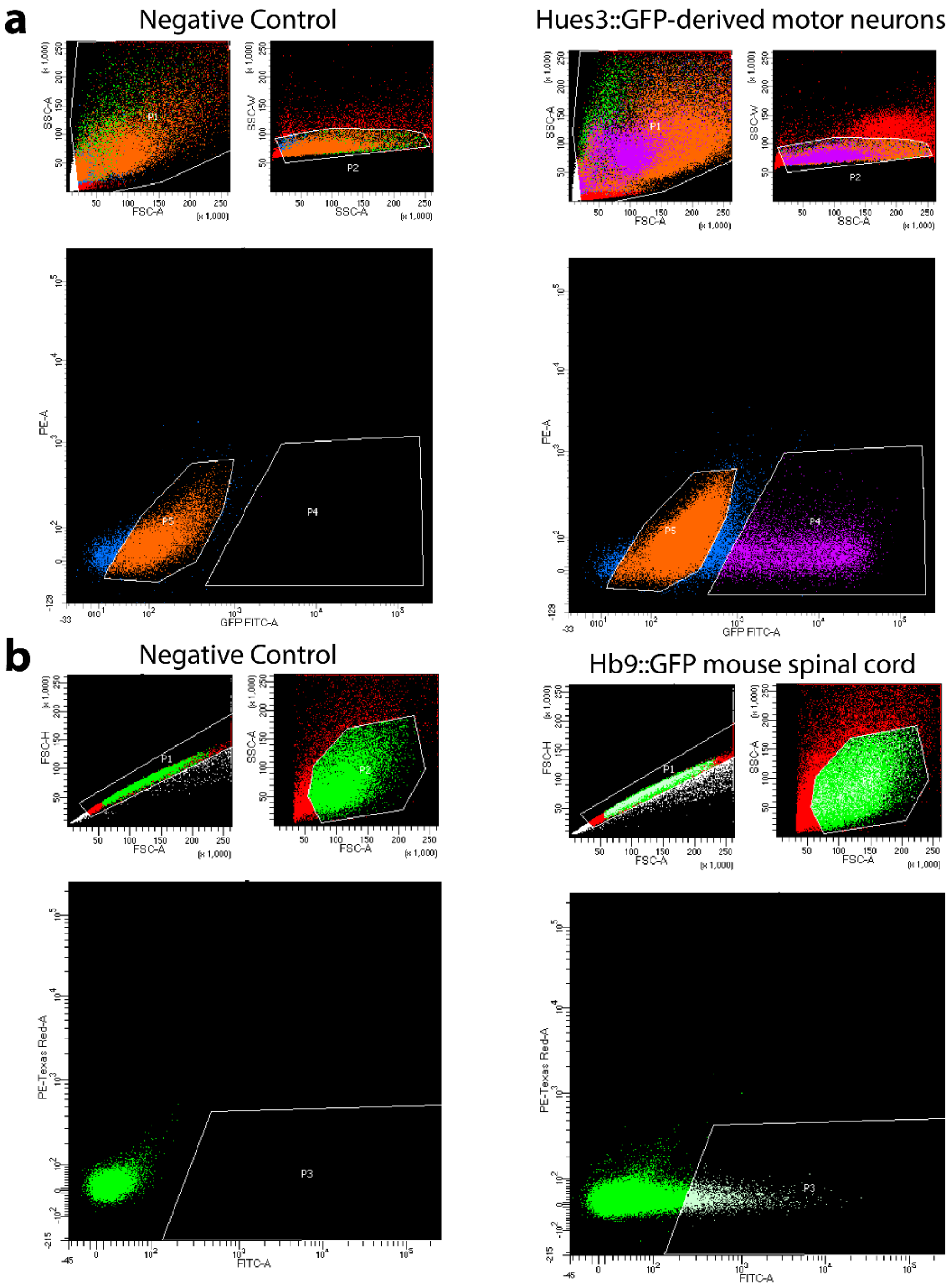

Supplementary Fig. 1 | Example of primary FACS gating parameters for mouse and human cultures. Examples of primary FACS gates for human (a) and mouse (b) cultures. *Left* Negative control samples consisting of non-Hues3:GFP-derived motor neurons and Hb9:GFP littermate lacking GFP expression. *Right* GFP-expressing samples from Hues3:GFP-derived cultures and Hb9:GFP-positive tissue.

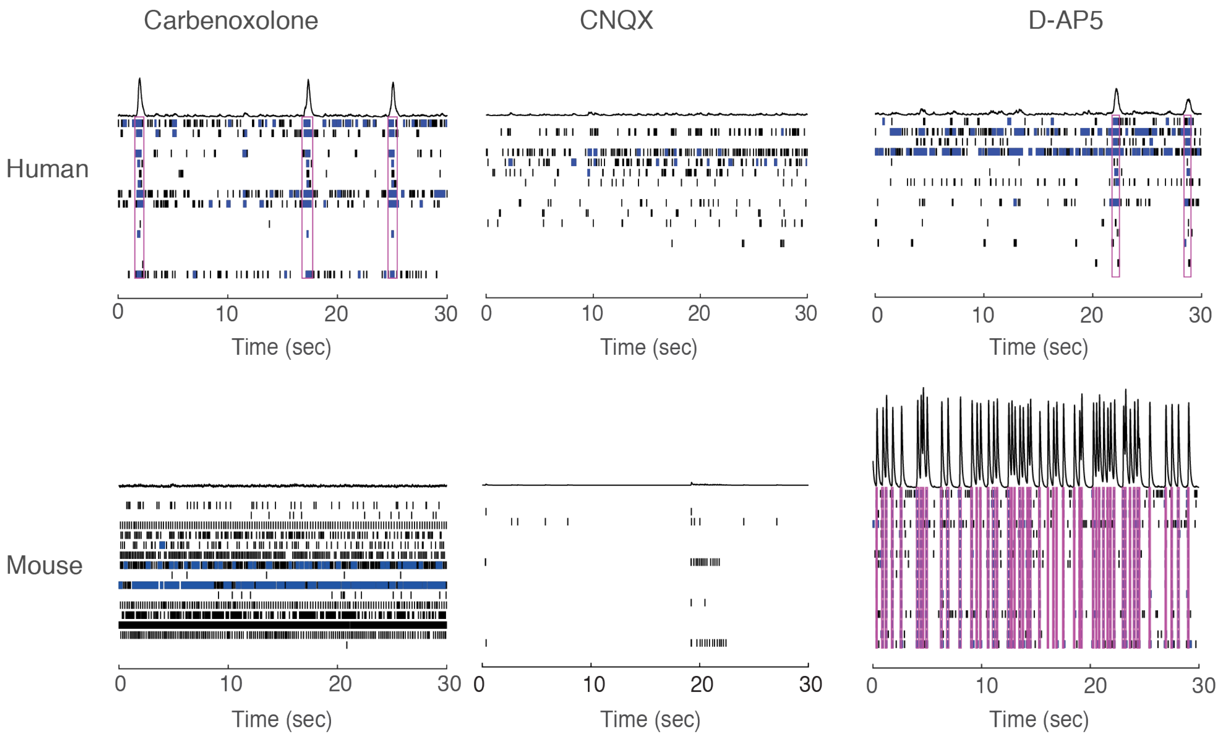

Supplementary Fig. 2 | Examples of effects of neurotransmitter blockers on network bursts. Raster plots show individual electrode spikes (black), within-electrode bursts (blue), and network bursts (pink rectangle). Traces above raster plots show population spike histogram. Network bursts remain after carbenoxolone treatment in human but not mice motor neurons. CNQX fully eliminates network bursts in both human and mouse motor neurons. Network bursts remain after D-AP5 treatment in both human and mouse motor neurons.

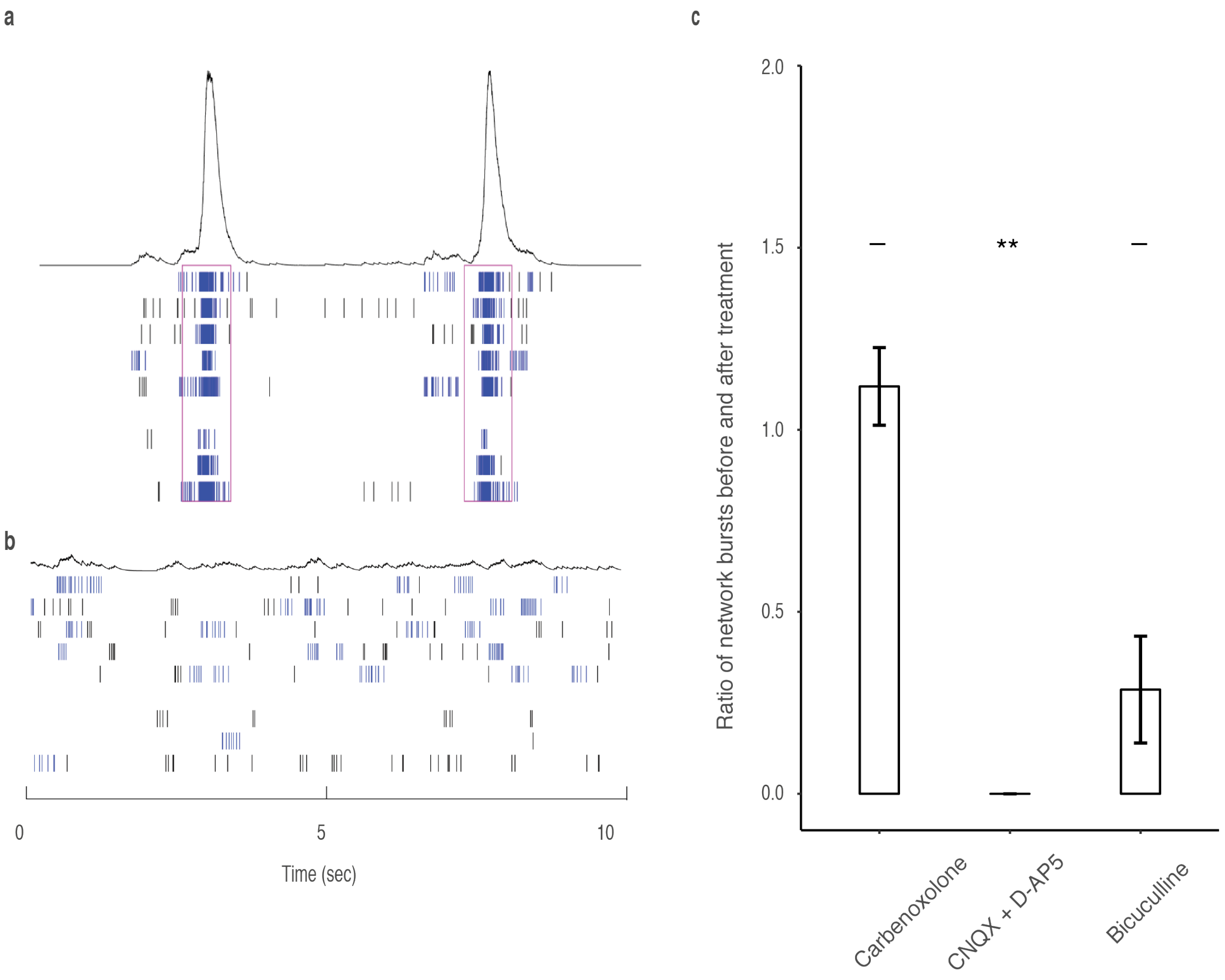

Supplementary Fig. 3 | Glutamate blockers eliminate network bursts in Islet::tdTomato hPSC-derived motor neurons. a, Sample raster plot before drug network blocker treatment. Trace above raster shows population spike histogram. **b**, Raster plot from the same MEA after CNQX and D-AP5 treatment. Trace above raster shows population spike histogram. **c**, Quantification of effects of transmitter blockers. P-values are represented as stars with the following notation: ≤ 0.0001 (****), ≤ 0.001(***), ≤ 0.01(**), ≤ 0.05 (*), and >0.05 (-).

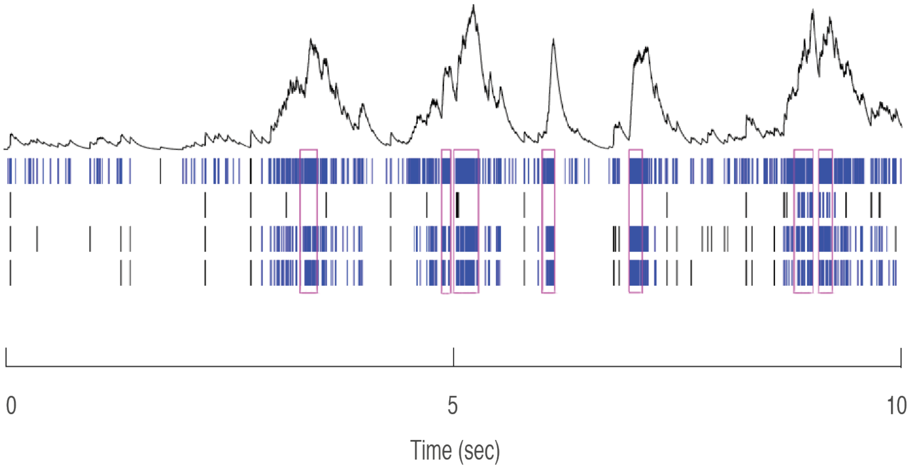

Supplementary Fig. 4 | Network bursts do not depend on the presence of co-cultured primary mouse glia. Raster plot from an MEA array shows network bursts in human FACS-purified PSC-derived motor neurons cultured without glia along with population spike histogram.

Supplementary Table 1 | Additional quantification of MEA activity at days 21-24

		Human		Mouse	
		Mean	SE	Mean	SE
**Electrode bursts**	Duration (s)	0.1806	0.0009	0.7177	0.0623
	Spikes/burst	12.9	0.1	75.8	6.4
**Network bursts**	Duration (s)	0.4028	0.0053	1.4717	0.0459
	Spikes/burst	174.6	2.33	1369.9	40.8
	Mean ISI (ms)	1.44	0.02	0.95	0.02

Supplementary Table 2 | Drug suppliers, concentrations, and targets.

Drug	Supplier (Cat. No.)	Concentration	Target	Reference
Mecamylamine	Tocris (2843)	100 µM	nACh receptor antagonist	22
CNQX	Tocris (0190)	15 µM	non-NMDA receptor antagonist	10
D-AP5	Tocris (0106)	25 µM	NMDA receptor antagonist	10
(+)-Bicuculine	Tocris (0130)	40 µM	GABA-A receptor antagonist	10
Carbenoxolone	Tocris (3096)	100 µM	Gap junction blocker	24
Nimodipine	Tocris (0600)	10 µM	L-type calcium channel blocker	27
Ω-conotoxin-MVIIA	Alomone Labs (C-670)	100 nM	N-type calcium channel blocker	28
TTA-A2	Alomone Labs (T-140)	10 µM	T-type calcium channel blocker	29
α-dendrotoxin	Alomone Labs (D-350)	100 nM	Kv1 channel blocker	32
XE991	Tocris (2000)	10 µM	Kv7 channel blocker	33
Linopirdine	Tocris (1999)	50 µM	Kv7 channel blocker	33
Guangxitoxin 1E	Tocris (5676)	100 nM	Kv2.1 and Kv2.2 channel blocker	34
E-4031	Tocris (1808)	10 µM	Kv11.1 channel blocker	35
Oxotremorine-M	Tocris (1067)	10 µM	Muscarinic agonist	33
Apamin	Tocris (1652)	100 nM	SK channel blocker	36
Charybdotoxin	Tocris (1087)	100 nM	BK channel blocker	36
ZD7288	Tocris (1000)	10 µM	HCN channel blocker	39
Riluzole	Tocris (0768)	10 µM	ALS therapeutic, Glutamate release inhibitor, persistent sodium channel blocker	42
Retigabine	Sigma-Aldrich (90221)	10 µM	Candidate ALS therapeutic, Kv7 channel opener	10,33
Mexiletine	Tocris (2596)	10 µM	Candidate ALS therapeutic, Sodium channel blocker	44
